# Effects of Selenium and Other Micronutrient Intake on Human Health

**DOI:** 10.3390/nu17132239

**Published:** 2025-07-07

**Authors:** Shuang-Qing Zhang

**Affiliations:** National Institute for Nutrition and Health, Chinese Center for Disease Control and Prevention, 27 Nanwei Road, Beijing 100050, China; zhangsq@ninh.chinacdc.cn; Tel.: +86-10-66237226

Since its discovery in 1817, selenium had long been considered toxic, until 1957, when the element was demonstrated to protect vitamin E-deficient rats against liver necrosis and recognized as an essential micronutrient. So far, a total of 25 human selenoprotein genes have been reported since the first mammalian selenoprotein (glutathione peroxidase 1) was identified in 1973. By virtue of selenoproteins, selenium plays physiological roles in the maintenance of homeostasis, regulation of transcription factors and apoptosis, control of the cellular redox state, development of the central nervous system, and immune and reproductive functions. Recently, selenium has obtained special attention in human diseases, such as cancers, diabetes mellitus, neurodegenerative dysfunctions, and aging [1]. Due to the narrow safe dose range of selenium intake and significantly different bioavailability of various selenium species, both selenium deficiency and selenium excess result in adverse effects [2]. A sufficient amount of proteins and amino acids, especially of serine, influences selenium status and selenoprotein biosynthesis [3]. This Special Issue of *Nutrients* explores the impact of selenium and the intake of other micronutrients on human health, providing a comprehensive understanding of selenium and other micronutrients in aspects of cells, animals, and humans.

Bai et al. found that the total selenium intake was the major factor in the blood selenium concentration in American adults besides gender, race, education status, income, body mass index, smoking, and alcohol status (Contribution 1). Animal studies have disclosed that selenium reinforces erythrocytes by decreasing osmotic fragility, that chromium harms erythrocytes by forming reactive oxygen species, and that manganese is highly related to erythrocytes via the modulation of iron metabolism. By analyzing American adults from the National Health and Nutrition Examination Survey 2015–2020, Costal et al. firstly found that selenium and manganese were positively associated with human erythrocytes, and that chromium was negatively correlated with human erythrocytes (Contribution 2). Sodium selenite and its nanoparticles showed contrasting effects on the skeletal muscle development of adolescent rats via the insulin signaling pathway, consistent with adipose tissue, indicating the potential therapeutic effects of selenite on muscle growth, including muscular dystrophies, cachexia, or sarcopenia (Contribution 3). The synergistic effect of selenium with coenzyme Q was found in mice with metabolic dysfunction-associated steatohepatitis from the reduction in oxidative stress and lipid peroxidation, as well as the suppression of ferroptosis, demonstrating the therapeutic potential of combined selenium and coenzyme Q for steatohepatitis and liver injury (Contribution 4). Selol (a mixture of selenitriglicerides) significantly increased the antioxidant enzyme activity in healthy mice and influenced the morphology of tumor cells in prostate tumor-bearing mice (Contribution 5). In their systematic review and meta-analysis, Costescu et al. found that Crohn’s disease patients had significantly lower serum magnesium levels and exhibited a lower magnesium intake; therefore, magnesium supplementation showed potential in alleviating Crohn’s disease by enhancing remission rates and sleep quality (Contribution 6). The cross-sectional study conducted in Romania implied the preventive potential of vitamin D against respiratory infections among preschool children, and supported the establishment of a public health strategy to recommend vitamin D supplementation for children without adequate exposure to sunlight (Contribution 7). Zhao et al. summarized the renal-protective effects and mechanisms of micronutrients from botanicals with medicine–food homology, and proposed botanical ingredients for the prevention and management of kidney diseases (Contribution 8). In summary, these studies shed light on the beneficial or detrimental effects of selenium and other micronutrients on human health, and provide reliable and convincing evidence for their research and development in future.

In my opinion, regarding selenium, special emphasis should be placed on the definite elucidation of the biological functions of poorly understood selenoproteins and the wide expansion of the well-defined biofunctions of selenoproteins. More novel selenium compounds and selenoproteins should be synthesized for human diseases such as cancers and neurological disorders.
